# µ-Opioid Receptor Gene (OPRM1) Polymorphism A118G: Lack of Association in Finnish Populations with Alcohol Dependence or Alcohol Consumption

**DOI:** 10.1093/alcalc/agt050

**Published:** 2013-05-30

**Authors:** Noora Rouvinen-Lagerström, Jari Lahti, Hannu Alho, Leena Kovanen, Mauri Aalto, Timo Partonen, Kaisa Silander, David Sinclair, Katri Räikkönen, Johan G. Eriksson, Aarno Palotie, Seppo Koskinen, Sirkku T. Saarikoski

**Affiliations:** 1 Department of Mental Health and Substance Abuse Services, National Institute for Health and Welfare, Helsinki, Finland; 2 Institute of Behavioural Sciences, University of Helsinki, Helsinki, Finland; 3 Institute of Clinical Medicine, Department of Medicine, University of Helsinki and Helsinki University Central Hospital, Helsinki, Finland; 4 Department of Psychiatry, South Ostrobothnia Hospital District, Seinäjoki, Finland; 5 Unit of Public Health Genomics, National Institute for Health and Welfare, and Institute for Molecular Medicine FIMM, University of Helsinki, Helsinki, Finland; 6 National Institute for Health and Welfare, Helsinki, Finland; 7 Department of General Practice and Primary Health Care, University of Helsinki, Helsinki, Finland; 8 Unit of General Practice, Helsinki University Central Hospital, Helsinki, Finland; 9 Folkhälsan Research Centre, Helsinki, Finland; 10 Institute for Molecular Medicine Finland, University of Helsinki, Helsinki, Finland; 11 Wellcome Trust Sanger Institute, Hinxton, Cambridge, UK; 12 Broad Institute of Harvard and MIT, Cambridge, USA; 13 Department of Health, Functional Capacity and Welfare, National Institute of Health and Welfare, Helsinki, Finland

## Abstract

**Aims:** The molecular epidemiological studies on the association of the opioid receptor µ-1 (OPRM1) polymorphism A118G (Asn40Asp, rs1799971) and alcohol use disorders have given conflicting results. The aim of this study was to test the possible association of A118G polymorphism and alcohol use disorders and alcohol consumption in three large cohort-based study samples. **Methods:** The association between the OPRM1 A118G (Asn40Asp, rs1799971) polymorphism and alcohol use disorders and alcohol consumption was analyzed using three different population-based samples: (a) a Finnish cohort study, Health 2000, with 503 participants having a DSM-IV diagnosis for alcohol dependence and/or alcohol abuse and 506 age- and sex-matched controls; (b) a Finnish cohort study, FINRISK (*n* = 2360) and (c) the Helsinki Birth Cohort Study (*n* = 1384). The latter two populations lacked diagnosis-based phenotypes, but included detailed information on alcohol consumption. **Results:** We found no statistically significant differences in genotypic or allelic distribution between controls and subjects with alcohol dependence or abuse diagnoses. Likewise no significant effects were observed between the A118G genotype and alcohol consumption. **Conclusion:** These results suggest that A118G (Asn40Asp) polymorphism may not have a major effect on the development of alcohol use disorders at least in the Finnish population.

## INTRODUCTION

Alcohol dependence and alcohol abuse are complex disorders involving multiple genetic and environmental factors. Based on twin and adoption studies, the hereditary component of alcohol dependence has been estimated to be 50–60% (e.g. Heath *et al*., 1997)*.*

One of the key neurotransmitter systems associated with alcohol-induced reward is the endogenous opioid system. It is known that alcohol consumption leads to an increase of endogenous opioids, β-endorphin in particular (Marinelli *et al*., 2003), which is linked to reinforcement after alcohol consumption. The binding of β-endorphin to µ-opioid receptors may reinforce alcohol drinking directly or indirectly through increased dopamine levels in the brain reward areas (Gianoulakis, 2009). Genetic variation in the μ-opioid receptor may hence affect the risk of developing alcohol use disorder by affecting the individual's response to alcohol. This hypothesis is supported by animal studies: e.g. mice lacking the μ-opioid receptor self-administer less alcohol than wild type mice (Roberts *et al*., 2000; Hall *et al*., 2001). Furthermore, opioid receptor antagonists such as naltrexone and naloxone reduce alcohol self-administration in animal studies, and in human studies they have been effective in preventing relapse to heavy drinking and in reducing craving for alcohol (reviewed, e.g. by Sinclair, 2001; Bouza *et al*., 2004; Thorsell, 2013). Taken together, accumulating evidence implicates human µ-opioid receptor as an important determinant in the reinforcing effects of alcohol, making it a good candidate gene in the search of the genetic variation influencing the development of alcohol use disorders.

Several single-nucleotide polymorphisms (SNP) in the opioid receptor µ-1 (*OPRM1*) gene have been identified, of which one in particular, SNP rs1799971 (=Asn40Asp, A118G), has been extensively studied with respect to addictive behavior. SNP rs1799971 is a missense variant in nucleotide 118 of the *OPRM1* cDNA resulting in an A to G substitution that causes amino acid asparagine (Asn) to be replaced by aspartic acid (Asp) in codon 40 (Bergen *et al*., 1997). The replacement was originally shown to result in 3-fold increase in β-endorphin binding and receptor activity *in vitro* (Bond *et al*., 1998). However, in more recent *in vitro* studies either the variant has shown more subtle increases in the binding affinity (Befort *et al*., 2001) or no differences have been observed between the variant and normal receptors (Beyer *et al*., 2004); some studies have even indicated that the 118G (Asp40) allele would result in decreased mRNA and protein levels (Heinz *et al*., 2005; Zhang *et al*., 2005).

Individuals with the 118G allele have been reported to be more sensitive to the stimulating and reinforcing effects of alcohol and to experience higher levels of craving for alcohol (Ray and Hutchison, 2004; van den Wildenberg *et al*., 2007; Miranda *et al*., 2010; Ray *et al*., 2010). It has been further postulated that a medication that reduces feelings of euphoria after alcohol consumption may be more successful among individuals with a genetic predisposition to greater feelings of euphoria (Ray and Hutchison, 2004). Indeed, in several studies the polymorphism has also been found to affect the efficacy of naltrexone and naloxone with the individuals carrying at least one G allele showing better responses, but also contradictory findings have been reported (reviewed by Chamorro *et al*., 2012).

The association between genetic variation of *OPRM1* and the response to alcohol is supported by results obtained by using animal models mimicking the A118G (Asn40Asp) variation. A recent study showed that in the brain areas central for drug reward, alcohol-induced dopamine release was restricted to individuals carrying the 118G allele (Ramchandani *et al*., 2011). The authors further confirmed these results by generating humanized mouse lines carrying the different alleles of the human *OPRM1* and performing brain microdialysis studies, which showed a 4-fold greater peak of dopamine response to an alcohol challenge in mice carrying the G allele when compared with mice with the A allele (Ramchandani *et al*., 2011). In rhesus monkeys, the *OPRM1* gene harbors a natural SNP polymorphism, C77G, that appears to be functionally similar to the human A118G polymorphism (Barr *et al*., 2004). The variant allele has been shown to exhibit similar physiological responses when compared with the human polymorphism including a 3.5-fold elevated affinity of the G77 allele encoded protein for β-endorphin (Miller *et al*., 2004) comparable with the 3-fold increased binding reported for the 118G allele in the original *in vitro* work (Bond *et al*., 1998). In line with the human observations, rhesus macaques carrying G77 variant showed increased alcohol-preference, consumed higher amounts of alcohol and drank to intoxication (Barr *et al*., 2004). Studies on rhesus monkeys also support the modulating role of the A118G polymorphisms on the naltrexone response: monkeys carrying the G allele showed greater reductions in alcohol intake after naltrexone administration when compared with the C allele carriers (Barr *et al*., 2010; Vallender *et al*., 2010).

The possible association between A118G polymorphism and different substance use disorders has been investigated in several studies (reviewed by van der Zwaluw *et al*., 2007 and Mague and Blendy, 2010). The results have been inconsistent. With respect to alcohol dependence, several case–control studies have reported significant associations or trends between alcohol dependence and the G allele (Rommelspacher *et al*., 2001; Bart *et al*., 2005; Nishizawa *et al*., 2006; Kim *et al*., 2009), whereas some groups have not found any association (Bergen *et al*., 1997; Sander *et al*., 1998; Gelernter *et al*., 1999; Kim *et al*., 2004a; Loh el *et al*., 2004; Zhang *et al*., 2006). Yet a few studies, including a recent study with a large study population, have reported the A allele as a risk allele (Town *et al*., 1999; Schinka *et al*., 2002; Du and Wan, 2009, Koller *et al*., 2012). In a meta-analysis including 10 studies with alcohol dependence as a main diagnosis, no significant association was found between the polymorphism and alcohol dependence (Arias *et al*., 2006), whereas a more recent meta-analysis including 12 studies, 5 conducted in Asians and 7 in Caucasians, indicated an association in Asians, but not in Caucasians (Chen *et al*., 2012). No association was observed in two family-based studies (Franke *et al*., 2001; Xuei *et al*., 2007). It has been speculated that the lack of association in many molecular epidemiological studies may result from small sample sizes, ethnic heterogeneity of the study populations or sample selection bias occurring due to the fact that the patients of the case–control studies generally consist of the treatment-seeking subpopulation of alcoholics, which may not be (genetically) representative of alcohol-dependent individuals in general (True *et al*., 1996; van der Zwaluw *et al*., 2007). Consequently, large population-based studies in homogeneous populations are warranted.

In the current study, we set out to analyze the possible association with the *OPRM1* A118G (Asn40Asp) polymorphism and both alcohol use disorders and alcohol consumption in three separate large general population-based cohorts of Finnish origin. The association between *OPRM1* polymorphism and alcohol use disorders was investigated in a study population comprising of 503 individuals with the DSM-IV diagnosis of alcohol dependence or alcohol abuse and their 506 age- and sex-matched controls. Our study population was drawn from a nationwide Health 2000 study cohort consisting of altogether 8028 individuals representative of the Finnish population. The Finnish population provides an excellent basis for genetic analyzes as it has remained genetically very homogeneous when compared with the majority of other populations of Caucasian origin. The other two general population-based cohort studies, FINRISK (*n* = 2360) and the Helsinki Birth Cohort Study (HBCS, *n* = 1393), lacked diagnosis-based information, but included detailed information on alcohol consumption. Molecular epidemiological studies on the association between *OPRM1* polymorphism and alcohol consumption, based on this kind of data, have not been published before.

## MATERIALS AND METHODS

### Study samples and measures of alcohol use

#### Health 2000 study population

The first study group consists of participants from the Health 2000 cohort study, which is a nationwide population-based health interview and examination survey carried out in Finland in 2000–2001. The study is described in detail elsewhere (Aromaa and Koskinen, 2004). The survey has been accepted by the ethics committees of the National Public Health Institute and the Helsinki and Uusimaa Hospital District, and all participants provided a written informed consent. The cohort includes 8028 individuals with age 30 or over. A total of 5955 participants donated blood samples and took part in a health status examination. Alcohol dependence and alcohol abuse as well as mood and anxiety disorders were assessed by using a diagnostic mental health interview, Munich-Composite International Diagnostic Interview (M-CIDI), yielding the diagnosis based on DSM-IV criteria (Diagnostic and Statistical Manual of Mental Disorders, 4th edn, American Psychiatric Association 2000). The subpopulation analyzed in the current study included all cases with a diagnosis of alcohol dependence or alcohol abuse (*n* = 512) and their sex- and age-matched controls having no mental disorders (*n* = 511). There were 99 women in both groups. Of the 512 cases, 414 (326 men/88 women) had a diagnosis of alcohol dependence, and 89 (78 men/11 women) had a diagnosis of alcohol abuse only. In addition, there were nine individuals with alcohol abuse diagnosis, but who lacked information concerning the dependence status; these cases were included in the analyses when considering the combined group of alcohol-dependent and alcohol abuse cases, but were excluded from the analyses focusing on the dependence and abuse subcategories. Genotyping results were obtained from 503 cases and 506 controls.

For analyses of alcohol consumption, values for the average intake of ethanol in pure ethanol gram per weight (kg) per week during the last 12 months were calculated from the available Health 2000 interview data. The analyses were made in the whole study population (*n* = 1006) and also separately among cases and controls.

#### FINRISK study population

The second study group consists of participants of the National FINRISK 2007 Study, which was carried out in Finland in 2007. The study was approved by the Coordinating Ethics Committee of the Hospital District of Helsinki and Uusimaa (229/EO/2006). Also the FINRISK was a nationwide health examination study based on the general adult population (Aalto *et al*., 2009). The sample was randomly selected from the Population Register Centre and was stratified according to sex, 10-year age groups and five geographical areas.

The whole FINRISK 2007 cohort included 10,000 individuals aged 25–74 years. A sub-sample of 4020 subjects received a questionnaire by mail that included questions regarding alcohol consumption, socio-demographic information, general health habits, chronic diseases and symptoms, as well as an invitation to a health check. A total of 2360 participants had both genotypic and phenotypic data available.

In the FINRISK 2007 Study, alcohol consumption was analyzed with three different methods: the Alcohol Use Disorders Identification Test (AUDIT) questionnaire, Timeline Follow-back (TLFB) and quantity-frequency (QF) questions. AUDIT has been developed to screen for heavy drinking (Saunders *et al*., 1993) and the instrument has 10 structured questions with the maximum score of 40. Heavy drinking is generally defined as having ≥8 points in the questionnaire (Reinert and Allen, 2007). According to this definition, 541 (23.0%) of the FINRISK study population would be classified as heavy drinkers. In this study, the AUD score was used as a continuous score.

The TLFB is a calendar-based interview in which subjects provide retrospective estimates of their daily alcohol consumption over a period of time prior to the interview (Sobell and Sobell, 1995). The interview was performed as described in more detail elsewhere (Aalto *et al*., 2009).The interviewers converted subjects' reported alcohol consumption volume into units of Finnish standard drinks. Heavy drinking can be generally considered as having a weekly average consumption of at least 14 Finnish standard drinks (one drink equivalent to 12 g of absolute alcohol) in the TLFB interview during the previous month. In the FINRISK population 166 (7.2%) individuals were classifiable as heavy drinkers according to these criteria. In the current study TLFB scores were analyzed as continuous measures.

In addition to analyses based on AUDIT and TLFB, the participants reported their alcohol use with two QF questions: (a) How many drinks usually have you had per drinking day during the past month? (b) How often usually per week have you used alcohol during the past month?

#### The Helsinki Birth Cohort Study cohort

The HBCS cohort is composed of 8760 individuals born between 1934 and 1944 in Helsinki University Central Hospital. Between 2001 and 2003, a randomly selected sample of 928 males and 1075 females participated in a clinical follow-up study with a focus on cardiovascular, metabolic and reproductive health, cognitive function and depressive symptoms. The study protocol was approved by the Institutional Review Board of the National Public Health Institute, and informed consent was obtained from all participants.

In the HBCS, alcohol use has been measured with a single item on the frequency of alcohol use (‘Have you recently used any alcoholic beverages; e.g. beer or spirit?’) with response options ranging from quitted completely (0), never used (1), less than once a month (2), 1–2 times a month (3), 1–2 times a week (4), three to seven times a week (5). Due to not knowing the reason for quitting alcohol consumption, the participants stating ‘Quitted completely’ were discarded from the analyses (*n* = 63). A total of 1393 participants had both genotypic and alcohol consumption data available. Of these, participants with X-chromosomal genotypes discrepant with the reported sex were removed from the analyses on alcohol consumption (*n* = 9). Altogether 1384 participants were thus included in the analyses on alcohol consumption.

### DNA genotyping

Genomic DNA was isolated from whole blood using standard procedures. For the Health 2000 study sample, A118G SNP (rs1799971, Asn40Asp) was genotyped using the fluorogenic 5′ nuclease assay method (TaqMan^®^) and pre-designed primers and probes (Taqman^®^ SNP Genotyping Assay C__8950074_1_, Applied Biosystems, Foster City, CA, USA) and Applied Biosystems 7300 Real Time PCR System (Applied Biosystems) according to manufacturer's protocols. In the FINRISK sample, genotyping was done using Sequenom MassARRAY System and iPLEX Gold chemistry (Sequenom, San Diego, California), according to the manufacturer's instructions, whereas the genotyping of the HBCS cohort was conducted with the Illumina 610k Quad Bead chip by the Wellcome Trust Sanger Institute.

### Statistical analyses

Analyses of Hardy–Weinberg equilibrium (HWE), genotype and allele frequencies, and association analysis between *OPRM1* rs1799971 genotypes and DSM-IV-based alcohol use disorder diagnosis or alcohol consumption were performed using the PLINK program, version 1.07 (http://pngu.mgh.harvard.edu/~purcell/plink/). Additive, dominant and recessive models were analyzed, controlling for age and sex, using linear or logistic regression models. Alcohol consumption was analyzed as a continuous variable. Health 2000 and FINRISK data were log-transformed to attain normality. When analyzing alcohol consumption in Health 2000 study population, the analyses were controlled for the alcohol use disorder diagnosis status.

### Power analyses

To estimate the power of our sample to detect a statistical association between the *OPRM1* genotype and DSM-IV-based diagnoses and alcohol consumption, a power calculation was performed using The Genetic Power Calculator (http://pngu.mgh.harvard.edu/~purcell/gpc/) (Purcell *et al.*, 2003) and the Quanto 1.2.4 software (http://hydra.usc.edu/gxe/). In all power calculations, type I error probability was set to 0.05 (two tailed). For the diagnosis-based phenotypes, a ≥80% statistical power to detect allele frequency differences was reached in the Health 2000 study with allele relative risk values of 1.4–2.0. For the consumption-related phenotypes, a ≥80% statistical power to detect associations was reached should the variant explain 0.35% (FINRISK with *n* = 2360) and 0.65% (HBCS with *n* = 1384) and 0.80% (Health 2000 with *n* = 1006) of the phenotypic variation.

## RESULTS

### Allele frequency

The *OPRM1* A118G (Asn40Asp, rs1799971) polymorphism was successfully genotyped in altogether 4762 samples (*n* = 1009 in Health 2000, *n* = 1393 in HBCS and *n* = 2360 in FINRISK). The background characteristics and *OPRM1* polymorphism genotypes of the Health 2000, FINRISK and HBCS population samples are shown in Table 1.

**Table 1.** Demographic characteristics and OPRM1 A118G genotype and allele frequencies in the three Finnish cohorts

	Health 2000	FINRISK	HBCS
	*n* (%)/mean (SD)	*n* (%)/mean (SD)	*n* (%)/mean (SD)
*n*	1009	2360	1393^a^
Men	812 (80.5%)	1078 (45.7%)	554 (39.8%)
Age (years)	46.4 (11.3)	50.6 (14.0)	63.4 (2.9)
A118G			
MAF(G)	20.8%	18.6%	19.0%
A/A	645 (63.9%)	1555 (65.9%)	916 (65.7%)
A/G	309 (30.6%)	734 (31.1%)	425 (30.5%)
G/G	55 (5.5%)	71 (3.0%)	52 (3.7%)

MAF, minor allele frequency; SD, standard deviation.

^a^Distribution of genotypes before applying quality control filters.

The frequency of the 118G (Asp40) allele was relatively similar in all three populations: 20.8% in Health 2000, 18.6% in FINRISK and 19.0% in HBCS (Table 1). The mean allele frequency for the G variant in all three populations combined was 19.2%. Expressed in terms of individuals having a particular genotype, of the total 4762 individuals tested, 65.4% were A/A, 30.8% were A/G and 3.7% were G/G. The genotypic distribution did not significantly differ from the expected Hardy–Weinberg distribution in any of the population samples (*P*-values >0.12).

### Diagnosis-based phenotypes

The association between *OPRM1* A118G (Asn40Asp, rs1799971) polymorphism and alcohol use disorders was analyzed in a population-based sample consisting of genotyping results from 503 individuals with a DSM-IV-based diagnosis of alcohol dependence or alcohol abuse and their 506 age- and sex-matched controls. No statistically significant associations were observed (Table 2, additive model; data not shown for recessive and dominant models). The prevalence of the 118 G allele was 21.2% among the controls and 20.3% among individuals with alcohol use disorders and the frequencies of AA, AG and GG genotypes were 63.2%, 31.0 and 5.7% among the controls and 64.6, 30.2 and 5.2% among the individuals with alcohol use disorders, respectively.

**Table 2.** Sex- and age-adjusted associations between OPRM1 A118G polymorphism and DSM-IV-based alcohol use disorder diagnosis

	Phenotype	*n*	OR^a^	95% CI		*P*-value
Health 2000	Alcohol dependence or alcohol abuse	1009^b^	0.95	0.77	1.17	0.60
	Alcohol dependence	915^c^	0.95	0.76	1.18	0.63
	Alcohol abuse	592^d^	0.94	0.63	1.39	0.75

Results obtained by using additive genetic model.

^a^Odds Ratios (ORs) were derived from the multiple logistic regression analyses.

^b^506 controls + 503 individuals with alcohol dependence and/or alcohol abuse diagnosis (including 8 individuals with alcohol abuse diagnosis but no information about dependence diagnosis).

^c^409 individuals with alcohol dependence + 506 controls.

^d^86 individuals with alcohol abuse + 506 controls

### Consumption-related phenotypes

The association with *OPRM1* polymorphism and alcohol consumption was analyzed in three different populations by using the data available from each group. For Health 2000 study population (*n* = 1006), the data was analyzed with respect to the average intake of ethanol in pure ethanol gram per weight kilogram per week during the previous year as calculated from the Health 2000 interview data. For the FINRISK study population (*n* = 2360) TLFB, AUDIT and QF-based information was used, whereas with respect to the HBCS study population (*n* = 1384) the analyses were based on drinking frequency information obtained from interview data. The results are shown in Table 3. No statistically significant associations were observed in any of the three cohorts (*P*-values >0.24) using either an additive genetic model (Table 3) or dominant or recessive models (data not shown). Also, no statistically significant results were obtained in the Health 2000 study population when the consumption was analyzed separately among controls (*n* = 504) and individuals with alcohol dependence/abuse diagnosis (*n* = 502) (data not shown).

**Table 3.** Sex- and age-adjusted associations between OPRM1 A118G polymorphism and alcohol consumption-related phenotypes in the three Finnish cohorts

	Phenotype	*n*	Beta^a^	95% CI		*P*-value
Health 2000	Average intake of ethanol in EtOH gram per kilogram per week during last year^b^	1006^c^	0.03	−0.08	0.13	0.63
FINRISK	AUDIT^b^	2350	0.02	−0.04	0.07	0.54
	QF^b^	2319	−0.04	−0.11	0.03	0.24
	TLFB^b^	2318	−0.02	−0.12	0.08	0.68
HBCS	Alcohol use frequency	1384	−0.03	−0.13	0.07	0.52

Results obtained by using additive genetic model.

AUDIT, Alcohol Use Disorders Identification Test; QF, quantity-frequency score; TLFB, Timeline Follow-back.

^a^Unstandardized betas were derived from the multiple regression analyses.

^b^Log-transformed values.

^c^Consumption data available from 504 controls and 502 individuals with alcohol use disorder. Data adjusted for diagnosis.

## DISCUSSION

The role of *OPRM1* A118G (Asn40Asp) polymorphism in susceptibility to alcohol use disorders and alcohol consumption has remained controversial in prior research. The polymorphism has in several studies been shown to affect alcohol sensitivity and consumption in animal models and in humans. Furthermore, μ-opioid antagonists have reduced alcohol consumption in both experimental setting and in the clinical use. However, molecular epidemiological studies investigating the association between the polymorphism and alcohol dependence have produced inconsistent results.

In order to clarify the role of *OPRM1* polymorphism in alcohol use disorders, we investigated the possible association of *OPRM1* A118G polymorphism on alcohol consumption and on susceptibility to alcohol dependence in three large population-based study populations. Our study populations included two cohorts of sizes 2360 and 1384, which were analyzed with respect to alcohol consumption as well as a diagnosis-based population sample of 503 individuals with alcohol dependence or alcohol abuse diagnosis and their 506 age- and sex-matched controls, which were drawn from a nationwide study cohort. To our knowledge, our study is the first cohort-based study investigating the association between *OPRM1* A118G polymorphism and alcohol use disorders or alcohol consumption.

Our study did not reveal any statistically significant association between alcohol dependence or alcohol abuse and A118G polymorphism (*P* > 0.05). The finding is in line with a number of previous studies (Bergen *et al*., 1997; Sander *et al*., 1998; Gelernter *et al*., 1999; Kim *et al*., 2004a; Loh el *et al*., 2004; Zhang *et al*., 2006). While there have been several findings suggesting an association, the results have been conflicting. A few studies have reported either a significant association (Bart *et al*., 2005; Nishizawa *et al*., 2006) or a non-significant trend (Rommelspacher *et al*., 2001; Kim *et al*., 2004b) between the G allele and alcohol dependence. In contrast, some studies have reported a significant association between the A allele and alcohol dependence (Town *et al*., 1999; Schinka et al., 2002; Du and Wan, 2009; Koller *et al*., 2012). Since the majority of the results pointing towards an association between the A118G polymorphism and alcohol dependence do not reach statistical significance and the few significant studies report conflicting results with respect to the ‘at-risk’ allele, the evidence regarding the link between *OPRM1* polymorphism and alcohol dependence risk can be considered scarce. This being said, however, the A118G (Asn40Asp) polymorphism seems to moderate the hedonic, but not the sedative, effects of drinking alcohol, as alcohol-dependent patients carrying the G-allele have greater alcohol-induced stimulation, vigor and positive mood (Ray *et al*., 2013), as well as a stronger link between desire to drink in the evening and later night drinking (Kranzler *et al*., 2013). Moreover, as G-allele carriers tend to have attenuated cortisol responses under a range of stressful events (Pratt and Davidson, 2009; Schwandt *et al*., 2011), it might modify their vulnerability to alcohol use disorders, or to anxiety disorders, or to depressive disorders, or to some combination thereof, known to be clinically relevant and prevalent (Pirkola *et al*., 2005).

Various variables can be used to measure the consumption of alcohol. In the current study several such variables have been used depending on the information available for the three study populations, i.e. average intake of ethanol in pure ethanol gram per kilogram per week, TLFB, AUDIT and QF as well as drinking frequency information obtained from interview data. No statistically significant association was observed with any of these different variables analyzed.

The prevalence of the 118G (Asp40) allele shows considerable variation between different ethnic populations (reviewed by Arias *et al.*, 2006; van der Zwaluw *et al*., 2007). Lowest frequencies have been observed in individuals with African origins (<5%) and the highest frequencies in Asians (25–45%). In Caucasian populations the frequency has been reported to be 10–15% (reviewed by Arias *et al*., 2006; van der Zwaluw *et al*., 2007). Compared with these previous results obtained from the Caucasian populations, the frequency in the Finnish study populations analyzed in the current study was high, ∼19%. The results were similar in all three cohorts analyzed, i.e. 20.8, 18.6 and 19.0%. The populations were genotyped with different reliable methods (Taqman, Sequenom and lllumina chip technologies) and so erroneous frequencies obtained due to genotyping errors are highly unlikely. In a previous study testing Finnish subjects the frequency of the 118G allele was considerably lower, i.e. 11.1% in unaffected controls and 13.6% among alcohol-dependent individuals (Bergen *et al*., 1997). There are no explicit explanations for such big discrepancies between the studies. However, in the study of Bergen *et al*., the study population consisted of 324 males of which 165 were criminal offenders undergoing forensic psychiatric examination and 159 were volunteers recruited through newspaper advertisement. Eighty-eight individuals belonging to both criminal offenders and control groups were diagnosed as alcohol dependent. As several studies have suggested an association between the *OPRM1* polymorphism and personality traits (Corley *et al.*, 2008; Luo *et al*., 2008; Troisi *et al*., 2011), a study where both alcohol-dependent and control populations are largely composed of criminal offenders may exhibit distorted genotype distributions.

The present study addresses a number of problems that have been encountered in association studies on alcohol dependence. In many case–control studies, the data have been collected among patients who have sought or received treatment for alcohol addiction thus representing a selected subpopulation of alcohol-dependent individuals. Many studies are also hampered by significant phenotypic variation and heterogeneity as well as ethnic diversity and insufficient size of study populations. In the current study, all subjects were diagnosed using the same diagnostic DSM-IV codes. The population-specific dataset is large, with age- and gender-matched cases (*n* = 503) and controls (*n* = 506) allowing detection of relative risk >1.4. The Finnish population provides an excellent basis for genetic analyzes as it has remained genetically very homogeneous when compared with the majority of other populations of Caucasian origin. We aimed to further limit the ethnic variation by excluding persons with a mother tongue other than Finnish or Swedish (the two official languages in Finland). With respect to analyses on alcohol consumption, the populations were of substantial size thus giving enough power to detect even relatively small effects. Based on the power calculations we should have been able to detect effects that would explain <1% of the variation in alcohol consumption. The results showing a lack of significant association between alcohol consumption and *OPRM1* polymorphism can thus be considered reliable, even though the variables used to estimate the alcohol consumption varied between the populations analyzed.

The major limitation of our study is the lack of knowledge on the different types of alcohol dependence in the subjects analyzed. Namely, alcohol dependence can be divided into several clinically and etiologically different subgroups, the main divisions being the Type 1 alcoholism characterized with later onset and little or no antisocial behavior, and Type 2 alcoholism, illustrated by an early onset, and a more severe course with higher levels of alcohol-related problems (Cloninger, 1987). In a Swedish case–control study analyzing Cloninger type 1 and type 2 alcoholics, the *OPRM1* 118G allele was associated with alcohol dependence, but no difference was observed in A118G genotype distribution between type 1 and type 2 alcoholics (Bart *et al*., 2005). However, further research is warranted to clarify the possible association between A118G polymorphism with the specific alcohol dependence subgroups.

To conclude, our fairly large cohort-based material (503 individuals with diagnosis of alcohol dependence or abuse and their 506 age- and sex-matched controls) does not support any association between the *OPRM1* A118G polymorphism and alcohol dependence. Neither was there an association between the polymorphism and alcohol consumption in any of the three study cohorts. Our results thus fail to clarify the seemingly controversial situation with accumulating proof indicating that *OPRM1* A118G polymorphism modifies alcohol-seeking behavior in experimental settings and moderates pharmacological and therapeutic responses, yet does not appear to be associated with risk of developing the alcohol use disorders in molecular epidemiological studies.

## Funding

Helsinki Birth Cohort Study has been supported by grants from the Academy of Finland, the Finnish Diabetes Research Society, Folkhälsan Research Foundation, Novo Nordisk Foundation, Finska Läkaresällskapet, Signe and Ane Gyllenberg Foundation, University of Helsinki, Ministry of Education, Ahokas Foundation, Emil Aaltonen Foundation. *Conflict of interest statement.* None declared.
